# Immunoreactivity for prostate specific antigen and Ki67 differentiates subgroups of prostate cancer related to outcome

**DOI:** 10.1038/s41379-019-0260-6

**Published:** 2019-04-12

**Authors:** Peter Hammarsten, Andreas Josefsson, Elin Thysell, Marie Lundholm, Christina Hägglöf, Diego Iglesias-Gato, Amilcar Flores-Morales, Pär Stattin, Lars Egevad, Torvald Granfors, Pernilla Wikström, Anders Bergh

**Affiliations:** 1 Departments of Medical Biosciences, Pathology, Umeå University, Umeå, Sweden; 2 Department of Urology, Institute of Clinical Sciences at Sahlgrenska Academy, University of Gothenburg, Gothenburg, Sweden; 3 Department of Drug Design and Pharmacology, Faculty of Health and Medical Sciences, University of Copenhagen, Copenhagen, Denmark; 4 Department of Surgical Sciences, Uppsala University, Uppsala, Sweden; 5 Department of Pathology and Cytology, Karolinska University Hospital, Stockholm, Sweden; 6 Department of Urology, Central Hospital, Västerås, Sweden

**Keywords:** Prognostic markers, Prostate cancer

## Abstract

Based on gene-expression profiles, prostate tumors can be subdivided into subtypes with different aggressiveness and response to treatment. We investigated if similar clinically relevant subgroups can be identified simply by the combination of two immunohistochemistry markers: one for tumor cell differentiation (prostate specific antigen, PSA) and one for proliferation (Ki67). This was analyzed in men with prostate cancer diagnosed at transurethral resection of the prostate 1975–1991 (*n* = 331) where the majority was managed by watchful waiting. Ki67 and PSA immunoreactivity was related to outcome and to tumor characteristics previously associated with prognosis. Increased Ki67 and decreased PSA were associated with poor outcome, and they provided independent prognostic information from Gleason score. A combinatory score for PSA and Ki67 immunoreactivity was produced using the median PSA and Ki67 levels as cut-off (for Ki67 the upper quartile was also evaluated) for differentiation into subgroups. Patients with PSA low/Ki67 high tumors showed higher Gleason score, more advanced tumor stage, and higher risk of prostate cancer death compared to other patients. Their tumor epithelial cells were often ERG positive and expressed higher levels of ErbB2, phosphorylated epidermal growth factor receptor (pEGF-R) and protein kinase B (pAkt), and their tumor stroma showed a reactive response with type 2 macrophage infiltration, high density of blood vessels and hyaluronic acid, and with reduced levels of caveolin-1, androgen receptors, and mast cells. In contrast, men with PSA high/Ki67 low tumors were characterized by low Gleason score, and the most favorable outcome amongst PSA/Ki67-defined subgroups. Men with PSA low/Ki67 low tumors showed clinical and tumor characteristics intermediate of the two groups above. A combinatory PSA/Ki67 immunoreactivity score identifies subgroups of prostate cancers with different epithelial and stroma phenotypes and highly different outcome but the clinical usefulness of this approach needs to be validated in other cohorts.

## Introduction

Independent transcriptomic studies have shown that prostate tumors can be categorized into three roughly equally common molecular subtypes with different clinical behavior [1, 2]. The subtypes were named prostate cancer subtype 1–3 (PCS1-3) in [1] and luminal A, luminal B, and basal cell like in [2]. PCS1 and luminal B cancers appeared similar and were characterized by high cell proliferation, poor differentiation, and prognosis [1, 2]. PCS2/luminal A cancers showed signs of luminal differentiation and had a favorable outcome. PCS3 resembled basal-cell-like cancers and were enriched for processes involving extracellular matrix organization, inflammation, and cell migration [1, 2]. Cell proliferation was considerably higher in PCS1/luminal B than in PCS2/luminal A, whereas the opposite was seen for tumor cell differentiation [1, 2]. In the prostate, cell proliferation and differentiation are mutually exclusive processes [3] both driven by androgens, but in a highly context dependent way [4].

By exploring the global gene-expression pattern and proteome in bone metastases, we have found that also metastases can be separated into molecular clusters, characterized by different biological processes, morphology, and clinical behavior [5–7]. One metastasis cluster showed gene-expression pattern recognizable from PCS2/luminal A, while two other metastasis clusters showed gene-expression patterns similar to PCS1/luminal B and PCS3/basal-cell-like tumors, respectively [1, 2, 6, 7]. Importantly, the luminal A like subtype showed signs of luminal cell differentiation and secretory activity (monitored for example by tumor cell PSA synthesis) and low cell proliferation, a phenotype similar to that of the normal prostate epithelium, while the luminal B-like showed signs of luminal cell dedifferentiation, low PSA expression, and high cell cycle activity [6, 7]. Collectively, these observations in primary tumors and metastases suggest that clinically relevant subgroup of prostate cancer can be defined by exploring markers of cell differentiation and cell proliferation.

Tumors from an historical cohort of prostate cancers diagnosed after transurethral resection of the prostate were therefore categorized by differences in their PSA and Ki67 immunoreactivity. Using different cut-offs for high and low immunoreactivity, the PSA/Ki67-defined groups were related to outcome in patients managed by watchful waiting and to previously collected data related to biological characteristics of these tumors. As previous studies in animal models and in tissue samples from patients have suggested that prostate tumors influence the surrounding non-malignant parts of the prostate in ways related to tumor aggressiveness, we also examined how the surrounding parts of the prostate were affected/colored/tinted (TINT = tumor instructed/indicating normal tissue), for additional description and references see [8–11].

## Materials and methods

### Patients and data collection

Data obtained from morphological analysis of full sections or tissue microarrays were available from a historical cohort of 419 men with prostate cancer [12, 13], detected after transurethral resection of the prostate due to voiding symptoms, 1975–1991, in Västerås, Sweden. Patients with symptomatic metastases were treated with androgen deprivation therapy, a few patients were treated with radiation or radical prostatectomy, while a majority of men were followed with expectancy (‘‘watchful waiting’’) according to clinical practice at that time (*n* = 307), for details see [13]. The cause of death was assessed by evaluation of medical records. For this cohort the cause-specific survival showed a close resemblance with relative survival indicating that cause of death has a high validity [14]. All cases were Gleason regraded by one pathologist [14]. At follow up in 2003, 37 (8.8%) of the men in the entire cohort were still alive, 156 (37%) had died from prostate cancer, 221 (53%) had died from other causes, and 5 (1.2%) had unknown outcome [13]. The median follow up of all patients was 19 years and the minimum follow up of those still alive at last follow up was about 12 years.

The tissue microarrays, containing at least 5 cores of tumor and 4 cores of matched non-malignant tissue from each patient, have previously been analyzed by immunohistochemistry for multiple biomarkers of potential prognostic significance (see Table 2 for references) and evaluated in relation to tumor characteristics and long-term clinical outcome. Data for tumor cell proliferation (fraction of Ki67 positive cells) were available for most of the cases (*n* = 389) [12, 15]. The available original tissue blocks were now sectioned and stained for PSA (*n* = 347), as earlier described [16], resulting in combined Ki67 and PSA data in 332 cases. The PSA staining was quantified using a scoring system based on the percentage (0: no staining, 1: 1%–25%, 2: 26%–50%, 3: 51%–75%, and 4: 76%–100%) and intensity (0: negative, 1: week, 2: moderate and 3: intense) of tumor epithelial cells stained. An immunoreactivity score was obtained by multiplying the scores for distribution and intensity, giving scores in the range of 0–12, as earlier described [16]. By using the combinatory PSA and Ki67 staining data, patients were categorized into 4 different groups: (1) PSA high/Ki67 low, (2) PSA high/Ki67 high, (3) PSA low/Ki67 low, and (4) PSA low/Ki67 high, in ways described below.

### Ethics statement

The tissue was collected as part of routine clinical practice and according to Swedish regulations at a time when informed consent was not required. The research ethical committee at Umeå university hospital (Regional Ethical Review Board in Umeå) approved of the study and waived the need for consent.

### Statistics

Cancer-specific survival was the main outcome in this study. Follow up started at date of the transurethral resection of the prostate and ended at date of death or at the end of follow up. Survival analysis was performed to investigate cancer-specific survival in men managed by watchful waiting in groups based on immunoreactivity for PSA and Ki67. Receiver operating characteristic curve analysis was performed to evaluate sensitivity and specificity for different cut-offs in identifying events of cancer-specific deaths. Cox regression analysis and Kaplan–Meier analysis with the log-rank test were used to test for statistical differences in cancer-specific survival between groups. Men who died of other causes were censored at the time of death. Continuous variables were given as median (25^th^; 75^th^ percentiles) and non-parametric statistics were used (Mann–Whitney *U* test, Spearman rank correlation). Ordinal variables were given as number (percentage) and non-parametric statistics was used (Chi-squared test and Spearman rank correlation). All tests were two sided and *P*-value less than 0.05 were considered statistically significant. Statistical analysis was performed using the SPSS 23.0.0 software for Os X (SPSS Inc., Chicago, IL, USA).

## Results

### Reduced tissue PSA and increased Ki67 immunoreactivity is related to poor outcome

**Fig. 1:**
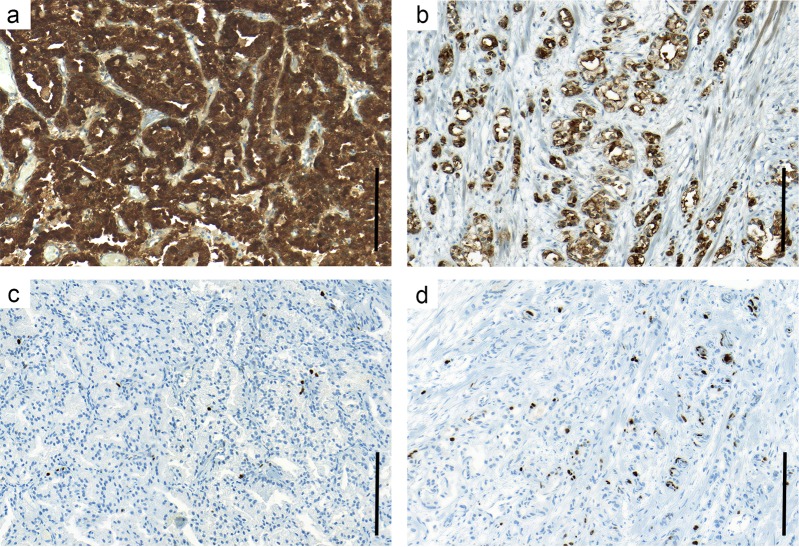
Representative tissue sections from two patients showing immunostaining for PSA and Ki67. One patient was characterized by high PSA immunoreactivity (**a**) showing uniform staining of high intensity in epithelial cells and a low fraction of Ki67 stained cells (**b**), and the other showed low PSA immunoreactivity (**c**) with a heterogeneous staining pattern of reduced intensity and a high fraction of Ki67 positive cells (**d**). Bar indicates 100 µm

In non-malignant prostate tissue the glandular luminal cells showed intense PSA staining (score 3) in at least 75% of the glandular tissue (score 4), resulting in a PSA immunoreactivity score of 12. This staining pattern was the most common also in prostate cancers (Fig. 1a), seen in 48% of the cases. However, in many men reduced PSA staining was seen in parts of or in the entire tumor, giving a PSA score < 12 (Fig. 1b).

Men managed with watchful waiting and available immunohistochemical data for PSA (*n* = 247) and/or Ki67 (*n* = 286) were analyzed for cancer-specific survival (for raw data, see Suppl. Table S1). As can be seen in Suppl. Fig. S1, both PSA and Ki67 provided prognostic information over a broad range of immunoreactivity scores. The median PSA (9) and Ki67 (2.67%) scores of the whole cohort were close to the optimal cut-off values for prognosticating death from prostate cancer in the part managed by watchful waiting (Suppl. Fig. S1), and, therefore, further used as cut-off values in Kaplan–Maier survival analysis. In an attempt to specifically enrich for aggressive prostate cancer cases, we also explored the 75^th^ percentile for Ki67 (quartile 4, 5.39%) as cut-off value.

**Fig. 2:**
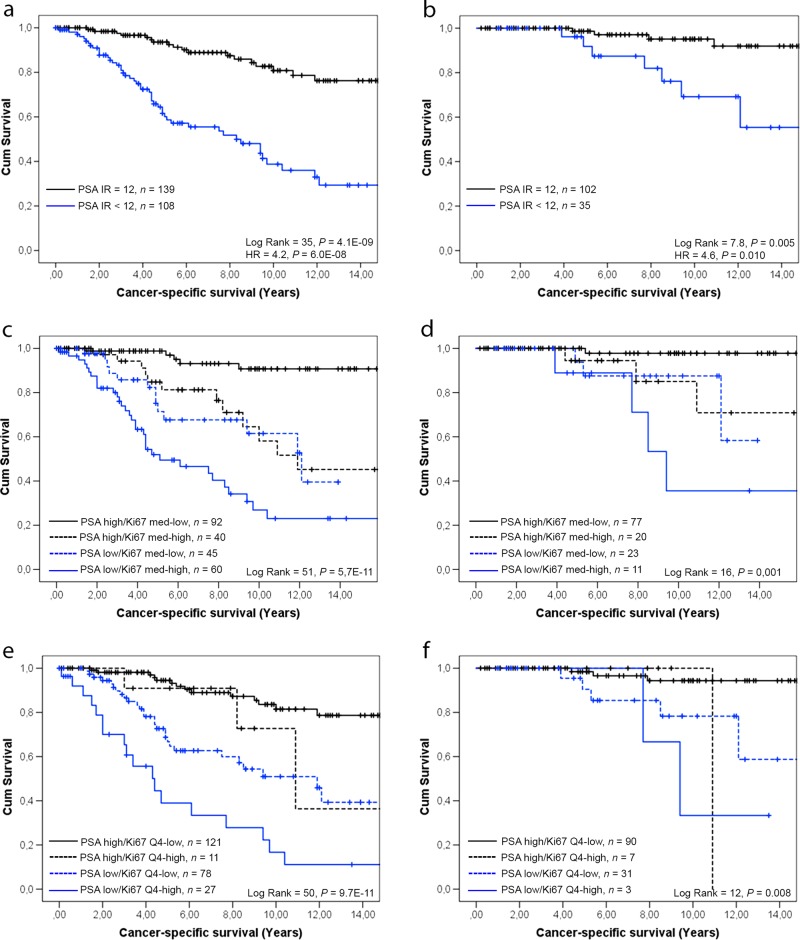
Kaplan–Meier survival analysis of PSA immunoreactivity (**a**, **b**) and a combinatory immunoreactivity score for PSA and Ki67 (**c**–**f**) in relation to cancer-specific survival of patients diagnosed at transurethral resection of the prostate and managed by watchful waiting. **a**, **c**, **e** All patients in the cohort and **b**, **d**, **f** Patients diagnosed with Gleason score <7 6 tumors. PSA immune reactivity (IR) was dichotomized by the median value 9 as high (score = 12) or low (<12). Ki67 was dichotomized by cut-off value for the median (**c**, **d**) as Ki67 median-high (Ki67 med-high ≥ 2.7%) or Ki67 med-low (<2.7%) or for the highest quartile (**e**, **f**) as Ki67 quartile 4-high (Ki67 Q4-high ≥ 5.4%) or Ki67 Q4-low (<5.4%)

Patients with a low tumor PSA score (median and below, i.e., < 12) had a short cancer-specific survival compared to those with high PSA (12) (Fig. 2a). Notable, low level of PSA staining was associated with poor prognosis also in men with Gleason score ≤ 6 (Fig. 2b). In contrast, men with high tumor Ki67 (above median, i.e., ≥ 2.7%) and particularly in the highest quartile (quartile 4, ≥ 5.4%) were associated with a poor outcome as earlier described in more detail [12, 15].

### Combined analysis of PSA and Ki67 immunoreactivity identifies patients with different prognosis

The tumor Ki67 and PSA immunoreactivity scores were inversely correlated in men managed by watchful waiting (*R*_S_ = −0.46, *p* < 0.001, *n* = 237), and both variables provided independent prognostic information from Gleason score in multivariate Cox survival analysis (Table 1). The PSA and Ki67 values were therefore used in combination. We first used the median scores; PSA (<12) and Ki67 (≥2.7%), as cut-off values to separate tumors into four different groups. Kaplan–Meier survival analysis showed that these groups had different outcomes when managed by watchful waiting, with PSA high/Ki67 median-low being the most favorable and PSA low/Ki67 median-high the worst combination (Fig. 2c). This was true also for patients with Gleason score ≤ 6 (Fig. 2d). Patients with PSA high/Ki67 median-high and PSA low/Ki67 median-low cases showed intermediate prognosis.

**Table 1:** Multivariate Cox analysis of PSA and Ki67 immunoreactivity and Gleason score (GS) in relation to cancer-specific survival of patients diagnosed at TUR-P and managed by watchful waiting

	HR	95% CI		*P*
		Lower	Upper	
GS ≤ 6, *n* = 131	1			
GS = 7, *n* = 47	3.8	1.8	8.1	4.8E-04
GS ≥ 8, *n* = 59	6.7	3.2	14	4.9E-07
PSA IR = 12, *n* = 132	1			
PSA IR < 12, *n* = 105	2.1	1.1	3.7	0.017
Ki67 (%)	1.05	1.0	1.1	0.038

PSA immunoreactivity (IR) was dichotomized by the median value 9 as high (IR = 12) or low (<9). Fraction of Ki67 positive tumor cells was analyzed as a continuous variable

In order to identify a subgroup of patients with a particularly poor prognosis, we also split patients into PSA/Ki67 groups using quartile 4 (≥5.4%) as cut-off value for Ki67 high; PSA high/Ki67 quartile 4-low (121/237, 51%, of men managed by watchful waiting), PSA high/Ki67 quartile 4-high (11/237, 4.6%), PSA low/Ki67 quartile 4-low (78/237, 33%), and PSA low/Ki67 quartile 4-high (27/237, 11%). As anticipated, patients with PSA low/Ki67 quartile 4-high had the worst prognosis (Fig. 2e). Among the Gleason score ≤ 6 patients, PSA low/Ki67 quartile 4-high were very rare, but it was still obvious that reduced PSA and/or increased Ki67 levels were associated with poor prognosis (Fig. 2f).

Taken together, those results indicated that a combinatory PSA and Ki67 immunoreactivity score added prognostic information to Gleason score in prostate cancer patients managed by watchful waiting. Furthermore, the combinatory score seemed more specific than either individual marker in prognosticating death from prostate cancer (Fig. 2 and Suppl. Table S2). Notably, the cut-off values for defining PSA/Ki67 high/low could be adjusted with the purpose of increasing sensitivity or specificity, respectively (Fig. S1 and Table S2).

### Clinical and histopathological characteristics of tumors categorized by their PSA and Ki67 immunoreactivity

As the identified subgroups based on PSA and Ki67 immunostaining showed differences in clinical behavior, we then examined their characteristics in more detail (using all available cases irrespective of treatment, and the quartile 4 was used to define high Ki67). The most common group, PSA high/Ki67 quartile 4-low (141/331, 43% of all cases), contained tumors with an immunohistochemistry staining pattern similar to that of normal prostate glands, that is homogeneous and intense PSA staining and low cell proliferation. This group was characterized by low Gleason score, low tumor extent and stage, and low fraction of bone metastases at diagnosis (Tables 2, 3). Furthermore, they showed low values of various markers in the tumor epithelium and in the tumor stroma previously related to poor outcome in this patient cohort (Tables 2, 3). Although the PSA high/Ki67 quartile 4-low subgroup showed the best prognosis, still 18% of the men in this group died from prostate cancer (see below). Using the median Ki67 as cut-off a smaller (106/331) PSA high/Ki67 median-low group where only 12% died from prostate cancer was identified.

**Table 2:** Clinical and histopathological variables in patients stratified by differences in Ki67 and PSA immunostaining

	PSA high/Ki67 low	PSA high/Ki67 high	PSA low/Ki67 low	PSA low/Ki67 high
	(*n* = 141, 42%)	(*n* = 17, 5%)	(*n* = 105, 32%)	(*n* = 68, 20%)
Clinical markers				
Age	74 (69; 78)	75 (71; 79)	74 (69; 78)	75 (69; 82)
GS			***a	***a,***b
4–6	95 (67)	9 (53)	37 (35)	7 (10)
7	29 (21)	3 (18)	23 (22)	7 (10)
8–10	17 (12)	5 (29)	45 (43)	54 (79)
Tumor stage		*a	***a	***a, **b
T1	94 (67)	7 (41)	38 (36)	11 (16)
T2	35 (25)	5 (29)	31 (30)	20 (29)
T3–4	11 (7.8)	5 (29)	33 (31)	34 (50)
x	1 (0.7)	0	3 (3)	3 (4)
M stage		*a	**a	***a,**b
0	100 (71)	11 (65)	73 (70)	35 (51)
1	3 (2)	2 (12)	13 (12)	23 (34)
x	38 (27)	4 (24)	19 (18)	10 (15)
Cancer (%)	10 (7.5; 45)	30 (10;70)	60 (20; 85)***a	88 (50; 95)***a,***b
PC death (%)	26 (18)	5 (29)	51 (49)***a	50 (74)***a,**b
Tumor markers				
pEGF-R score (13) (epithelial)	3.1 (2.4; 3.6) (*n* = 110)	3.6 (3.1; 3.9) (*n* = 8)	3.3 (2.8; 3.6) (*n* = 83)	3.6 (3.3; 4.0) ***a,**b (*n* = 45)
ErbB2 score (28) (epithelial)	2.8 (2.0; 3.0) (*n* = 126)	3.0 (2.7; 3.8)**a (*n* = 14)	3.0 (2.3; 3.8)**a (*n* = 99)	3.0 (3.0; 4.0) ***a,*b (*n* = 63)
ERG (35)				
negative	105 (79.5)	10 (62.5)	43 (44.3)	21 (32.3)
positive (epithelial)	27 (20.5)	6 (37.5)	54 (55.7) ***a	44 (67.7) ***a
pAkt score (29) (epithelial)	2.6 (2.2; 2.9) (*n* = 109)	2.8 (2.5; 3.3) (*n* = 12)	2.8 (2.4; 3.1)**a (*n* = 81)	3.1 (2.8; 3.6) ***a,***b (*n* = 49)
Ki67 (%) (12, 15) (epithelial)	1.4 (0.4; 2.7) (*n* = 141)	8.8 (7.5; 13.6) ***a,***b (*n* = 17)	2.5 (1.2; 3.6)***a (*n* = 105)	10.9 (7.2; 15.6) ***a,***b (*n* = 68)
Vascular density (%) (12, 15)	11 (8; 16) (*n* = 138)	16 (9; 19) (*n* = 17)	15 (10; 21)**a (*n* = 101)	19 (12; 24)***a,*b (*n* = 68)
Hyaluronic acid score (32) (stroma)	7.1 (4.6; 9.0) (*n* = 139)	9 (6; 11)*a (*n* = 17)	7.8 (5.6; 9.8)*a (*n* = 105)	8.6 (6.2; 11.3)***a (*n* = 67)
Mast cell density (%) (343)	13 (9; 16) (*n* = 134)	14 (7; 17) (*n* = 16)	12 (8; 16) (*n* = 100)	8 (4; 13)***a,***b (*n* = 65)
Androgen receptor (%) (30) (stroma)	50 (39; 65) (*n* = 136)	52 (22; 67) (*n* = 16)	48 (28; 64) (*n* = 103)	37 (14; 55)***a,**b (*n* = 67)
Caveolin-1 score (31) (stroma)	3.0 (2.8; 3.4) (*n* = 139)	3.1 (2.9, 3.4) (*n* = 16)	3.0 (2.8; 3.3)*a (*n* = 101)	2.8 (2.6; 3.1) ***a,**b (*n* = 64)
CD163 (%) (33)	16 (11; 22) (*n* = 87)	21 (12; 30) (*n* = 4)	19 (16; 28)***a (*n* = 53)	19 (14; 26) (*n* = 29)
TINT markers				
Ki67 (%) (12, 15) (epithelial)	0.2 (0; 1.2) (*n* = 138)	0 (0; 1.3) (*n* = 17)	0.3 (0; 1.2) (*n* = 95)	0.5 (0; 2.5) (*n* = 57)
pEGF-R score (13) (epithelial)	3.0 (1.8; 3.5) (*n* = 111)	2.7 (2.1; 3.5) (*n* = 9)	3.3 (2.5; 3.8) **a (*n* = 79)	3.5 (3; 3.9) **a (*n* = 40)
pAKT score (29) (epithelial)	2.0 (1.5; 2.5) (*n* = 92)	2.0 (1.4; 2.3) (*n* = 11)	2.3 (1.6; 2.8) (*n* = 56)	2.4 (1.5; 2.8) (*n* = 34)
ERG (35)				
negative	117 (92.9)	13 (76.5)	75 (85.2)	40 (83.3)
positive (epithelial)	9 (7.1)	4 (23.5)*a	13 (14.8)	8 (16.7) *a
Hyaluronic acid score (32) (stroma)	6.3 (4.3; 8.0) (*n* = 135)	5.5 (3.8; 8.1) (*n* = 17)	6.5 (5.0; 9.0) (*n* = 99)	7 (5; 9) (*n* = 59)
Mast cell density (%) (34)	12 (8; 15) *n* = 130	12 (9; 16) (*n* = 17)	14 (10; 20) **a (*n* = 91)	14 (11; 20) **a (*n* = 53)

Continous variables given as median (25^th^;75^th^ percentiles), Ordinal variables given as number (percentage)

x = unknown

a = significantly different from PSA high/Ki67 low

b = significantly different from PSA low/Ki67 low

Mann Whitney *U* test or Chi square test, *p < 0.05, **p < 0.01, ***p < 0.001

**Table 3:** Significant Spearman rank correlations between tumor PSA score and Ki67 labeling index with other previously measured variables of prognostic significance (see Table 2 for references) describing tumor and surrounding normal prostate tissue (TINT) [37]

	Correlation coefficient for tumor PSA score	Correlation coefficient for tumor Ki67 labeling index
Clinical markers		
Gleason score	−0.54^c^ (*n* = 346)	0.50^c^ (*n* = 389)
Tumor stage	−0.41^c^ (*n* = 339)	0.42^c^ (*n* = 382)
M stage	−0.31^c^ (*n* = 272)	0.33^c^ (*n* = 301)
Cancer (%)	−0.47^c^ (*n* = 346)	0.45^c^ (*n* = 389)
Overall survival	0.21^c^ (*n* = 346)	−0.15^b^ (*n* = 389)
Tumor markers		
Ki67 (%)	−0.46^c^ (*n* = 331)	
pEGF-R score	−0.21^b^ (*n* = 252)	0.28^c^ (*n* = 293)
pAkt score	−0.31^c^ (*n* = 255)	0.36^c^ (*n* = 278)
ErbB2 score	−0.29^c^ (*n* = 307)	0.29^c^ (*n* = 350)
Vascular density (%)	−0.24^c^ (*n* = 330)	0.28^c^ (*n* = 381)
Hyaluronic acid score (stroma)	−0.18^b^ (*n* = 334)	0.27^c^ (*n* = 384)
Mast cell density (%)	0.21^c^ (*n* = 322)	−0.13^a^ (*n* = 362)
Androgen receptor (%) (stroma)	0.17^b^ (*n* = 329)	−0.17^b^ (*n* = 373)
PDGFR-beta (stroma) (37)	−0.15^a^ (*n* = 248)	0.21^c^ (*n* = 283)
Caveolin-1 score (stroma)	0.25^c^ (*n* = 326)	−0.25^c^ (*n* = 370)
CD163 (%)	−0.24^b^ (*n* = 177)	
Erg (positive or not)	−0.39^c^ (*n* = 315)	0.32^c^ (*n* = 350)
TINT markers		
Ki67		0.17^b^ (*n* = 360)
pEGF-R	−0.19^b^ (*n* = 244)	0.25^c^ (*n* = 284)
PDGFR-beta (stroma) (37)	−0.13^a^ (*n* = 302)	0.15^b^ (*n* = 344)
Hyaluronic acid score (stroma)	−0.14^a^ (*n* = 318)	0.14^b^ (*n* = 363)
Mast cell density (%)	−0.22^c^ (*n* = 299)	
Caveolin-1 score (stroma)		−0.11^a^ (*n* = 352)
Erg (positive or not)		0.16^b^ (*n* = 331)

^a^Correlation is significant at the <0.05 level (2-tailed)

^b^Correlation is significant at the <0.01 level (2-tailed)

^c^Correlation is significant at the <0.001 level (2-tailed)

The group most different from that above, defined by PSA low/Ki67 quartile 4-high (68/331, 21% of all cases) was characterized by high Gleason score, high tumor volume and stage, many cases with bone metastases already at diagnosis, and in this group 74% of the patient died from prostate cancer (Tables 2 and 3, Fig. 2). Several markers previously associated with poor outcome showed levels suggesting particularly aggressive disease in this group (Tables 2, 3). For example, the highest levels of pEGF-R, ErbB2, pAkt, and Erg as a marker for TRMPSS2-ERG fusion gene [17] were found in the tumor epithelium of this group. The tumor stroma showed signs of a reactive response [18, 19] with increased type 2 (CD163+) macrophage infiltration, vascular density and hyaluronic acid, and reduced levels of caveolin-1, androgen receptors, and mast cells (Tables 2, 3). All these tumor characteristics were seen also in the larger group (116/331) defined by PSA low/Ki67 median-high, a group where 66% of the men died from prostate cancer (data not shown).

The 2^nd^ largest group (105/331, 32%) contained cases defined by PSA low/Ki67 quartile 4-low. Also this group had higher Gleason score, tumor volume, stage, and fraction of cases with bone metastases at diagnosis compared to the PSA high/Ki67 quartile 4-low group (Table 2). They also had a less favorable outcome than the PSA high/Ki67 quartile 4-low group, but the prognosis was better than for the PSA low/Ki67 quartile 4-high group (Table 2, Fig. 2). Accordingly, markers previously found associated with a poor prognosis suggested that this group scored intermediate between the other groups. About 50% in this group died from prostate cancer (see below).

The group defined by PSA high/Ki67 quartile 4-high contained very few patients (17/331, 5%) suggesting that this phenotype is uncommon. This group of patients had higher tumor volume and stage and percentage of cases with bone metastases than the group with PSA high/Ki67 low, as well as significantly increased levels of ErbB2 and hyaluronic acid (Table 2).

We then examined whether the tumor instructed normal tissue response was associated with tumor subtype. Subgroups PSA high/Ki67 quartile 4-low and PSA low/Ki67 quartile 4-high, the groups with the best and worst prognosis, respectively, showed some morphological differences in the benign parts of the tumor bearing prostate. The benign parts of prostates carrying PSA low/Ki67 quartile 4-high tumors was characterized by significantly increased pEGF-R (*P*  < 0.01) in the epithelium and increased number of mast cells (*P* < 0.01) in the stroma (Table 2). Epithelial pAkt (*P* = 0.07) and Ki67 (*P* = 0.07) in benign glands, and hyaluronic acid in the stroma (*P* = 0.07) also tended to be increased.

As noted above disease outcome differed within each subgroup and we therefore compared patients dying from prostate cancer to those that died from other causes or were alive. In the PSA high/Ki67 quartile 4-low tumors, the relatively few cases that died from prostate cancer had higher median Gleason score (7 vs. 6, *P* < 0.001), tumor stage; (2 vs. 1, *P* < 0.05), tumor content (60 vs. 10%, *P* < 0.001), and Ki67 index (2.7 vs. 1.2%, *P* < 0.01). They also showed signs of a more pronounced stroma reaction with more hyaluronic acid (8 vs. 7, *P* < 0.05), and blood vessels (14 vs. 11, *P* < 0.05), as well as lower caveolin-1 in the tumor stroma (3 vs. 3, *P* < 0.05) than those alive or dying from other causes. In the group with PSA low/Ki67 quartile 4-low where 51% died from prostate cancer, the men who died from prostate cancer had higher Gleason score (8 vs. 6, *P* < 0.001), higher tumor volume (75 vs. 30 %, *P* < 0.01), higher stage (3 vs. 1, *P* <0.001), and more commonly metastases at diagnosis (25 vs. 3%, *P* < 0.01), but their PSA or Ki67 staining scores did not differ from those alive or dying from other causes. They also had higher hyaluronic acid staining in tumor stroma (9 vs. 7, *P* < 0.01), more tumor infiltrating CD163 + macrophages (25 vs. 19, *P* < 0.05), reduced stroma androgen receptors (42 vs. 52, *P* < 0.05) and reduced caveolin-1 (2 vs. 3, *P* < 0.05). The few patients dying from other causes in the PSA low/Ki67 quartile 4-high group had lower median GS (7 vs. 9, *P* < 0.01) than those dying from prostate cancer. In summary, standard prognostic markers like GS and the magnitude of stroma response affected prognosis within the PSA/Ki67 subgroups.

## Discussion

Recent studies have shown that localized prostate cancer [1, 2] and prostate cancer bone metastases [5–7] can be divided into highly different molecular subtypes. Subtype identity provided additional information to Gleason score regarding outcome, suggesting that subtype should be determined already at diagnosis. We therefore examined if some of the subtypes could be defined in a simpler way than by global gene-expression analysis, similar to what is regularly done in breast cancer diagnostics where clinically relevant subgroups are stratified by surrogate immunohistochemical markers [20]. In this paper PSA and Ki67 immunoreactivity was analyzed as surrogate markers for tumor cell differentiation and proliferation, respectively, and combined to differentiate prostate cancer patients managed by watchful waiting into subgroups with different prognosis and, furthermore, with different biological characteristics with therapeutic implications.

High Ki67 and low PSA immunoreactivity identified tumors, probably enriched for the PCS1/luminal B molecular subtypes [1, 2, 7], in patients with poor outcome when managed by watchful waiting. This finding was anticipated as high cell proliferation measured by Ki67 labeling [12, 15, 21–24] or by commercially available gene-expression tests such as Ciphergen, Oncotype, and Prolaris examining proliferation associated genes [25], is associated with aggressive disease. Low tumor cell PSA, although not commonly used as a biomarker, is associated with poor outcome in primary tumors [26] and in bone metastases [16], and patients with high Gleason score tumors with low serum PSA levels have a worse prognosis than those with high serum PSA [27]. In the current study, PSA staining could stratify patients with Gleason score 6 tumors into good prognosis (less than 5% cancer-specific death within 14 years) and patients with an almost 50% risk of death within 14 years.

The PSA low/Ki67 high group was enriched for cases with high Gleason score, advanced stage, early metastasis and, accordingly, poor prognosis, and probably identifies patients that irrespectively of Gleason score may need early active treatment. The association with increased pEGF-R [13], ErbB2 [28], and pAkt [29] signaling in the tumor epithelium, and with multiple signs of a particularly active tumor stroma response [18, 19], such as decreased stroma androgen receptors [30] and caveolin-1 [31], increased angiogenesis [15], high stroma hyaluronic acid [32] and a M2-macrophage (CD163+) [33] and mast cell- [34] associated inflammation suggest several potential targets that deserve future attention. This subgroup of tumors was apparently also associated with expression of the *TRMPSS2-ERG* fusion gene [35] and with changes in the so called tumor instructed normal tissue, see [8–11], although of moderate magnitude, separating it from prostates with less aggressive tumors.

In contrast, PSA high/Ki67 low staining identified a large group of men, with a good prognosis when managed with watchful waiting. This group of patients is probably enriched for the PCS2/luminal A molecular subtypes [1, 2, 7] and, having low Gleason score, low tumor volume, and seldom developed bone metastases, they may be suitable for active surveillance. On the other hand, almost 1 of 5 men in the group still died from prostate cancer suggesting that this group could be further divided into prognostic relevant subgroups. One way to do this is to use a lower cut-off for defining Ki67 high vs. low. Thus, our PSA high/Ki67 low tumors show clinical similarities to luminal A breast cancer as this large group of patients as a whole show a good prognosis but where a subgroup is metastatic and lethal [36, 37]. The patients dying from prostate cancer in the PSA high/Ki67 low group had higher Gleason score and a more reactive tumor stroma than the others.

A large group of patients in this study were characterized by low PSA and low proliferation. This group had morphological characteristics and clinical outcome intermediate between the other two common subtypes. It remains to be shown whether this group is enriched for PCS3/basal-cell-like tumors [1, 2, 7]. To explore this, we need to identify immunohistochemical markers specific for this subgroup. In this group ~50% died from prostate cancer. Those dying were characterized by higher Gleason score, more advanced tumor stage and M-status, but also by a more reactive tumor stroma.

Very few tumors showed homogeneously high PSA staining together with markedly raised cell proliferation. This suggest that prostate tumor development is associated with cellular dedifferentiation and reduced PSA synthesis prior to a major increase in cell proliferation.

This study has some weaknesses. It is based on historical samples from the pre-PSA era and it deals with cancers diagnosed at transurethral resection of the prostate that may be different from contemporary PSA and biopsy detected tumors. The Gleason scores used are not identical to the current 2014 standard, but probably sufficient to demonstrate that PSA/Ki67-defined subgroups have different Gleason scores and that Gleason score still matters within subgroups. The PSA scoring used is not optimal to describe heterogeneities in intensity and distribution. For example, cases with different staining patterns such as homogeneously low intensity may end up with the same score as high intensity in a restricted area, but it is probably sufficient (as in this study) to separate cases with a staining pattern similar to that in non-malignant prostate from those with any reduction in staining. When men managed with watchful waiting developed symptoms from metastases they were treated with androgen ablation, whereas those without symptoms received no further treatment. If such late treatment in some way prolonged life it would tend to underestimate differences between groups. For patients who received treatment directly after diagnosis different types of treatments were used and this may have affected survival (therefore these patients were excluded from survival analysis) and the fraction of patients dying from prostate cancer in groups listed in Table 2 may thus to some extent be affected by treatment.

In conclusion, primary prostate cancer can be separated into subgroups of clinical significance simply by PSA and Ki67 immunostaining, probably because this combination separates tumors in relation to cell differentiation and cell proliferation. By using different cut-offs for high and low PSA/Ki67 staining, the size of the subgroups and the sensitivity and specificity to detect cases with particularly good or bad prognosis can be varied. The subgroups defined by using the median PSA score and the highest quartile for Ki67 as cut-offs were related to standard prognostic markers such as Gleason score, tumor stage, and presence of metastases, but they also showed additional prognostic value and diverse biological characteristics similar to those recently described for the three molecular subgroups of prostate cancer [1, 2] and prostate cancer bone metastases [6, 7]. Additional studies of biopsy needle diagnosed cases are needed to evaluate the clinical usefulness of stratifying prostate cancers by their PSA/Ki67 staining characteristics, and furthermore to define the best scoring method and relevant cut-off values.

## Supplementary information

figure S1

table S1

table s2

Suplemental figure legend
