# Supplementary figures and images for: Immunoreactivity for prostate specific antigen and Ki67 differentiates subgroups of prostate cancer related to outcome
Source: Mod Pathol. 2019 Apr 12;32(9):1310–9. doi: 10.1038/s41379-019-0260-6 (PMC6760646; doi:10.1038/s41379-019-0260-6)

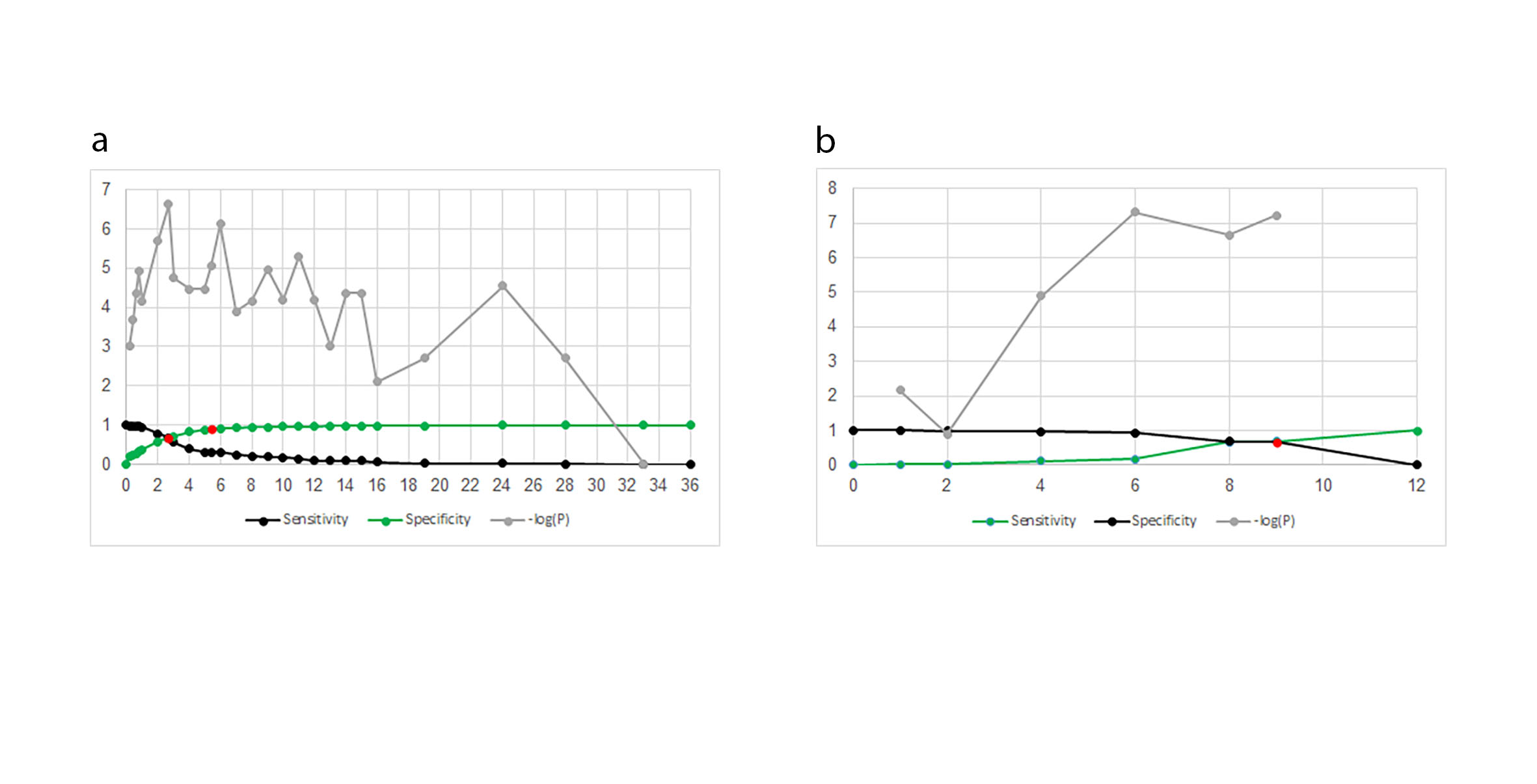

Supplement: Supplementary file 1 — figure S1 [file 41379_2019_260_MOESM1_ESM.jpg]
