# Supplementary material for: Immunoreactivity for prostate specific antigen and Ki67 differentiates subgroups of prostate cancer related to outcome
Source: Mod Pathol. 2019 Apr 12;32(9):1310–9. doi: 10.1038/s41379-019-0260-6 (PMC6760646; doi:10.1038/s41379-019-0260-6)
Supplement: Supplementary file 2 — table S1 [file 41379_2019_260_MOESM2_ESM.docx]

**Supplementary Table S1.** Data on survival, cause of death, Ki67 and PSA immunoreactivity (IR) score in individual patients managed by watchful waiting.

| **Patient** | **Follow-up time (years)** | **Cancer specific survival** | **Ki67 IR** | **PSA IR** |
| --- | --- | --- | --- | --- |
| 1 | 11,8 | 0 | 0,0 |  |
| 2 | 11,9 | 0 | 0,1 |  |
| 3 | 12,3 | 0 | 0,9 | 12 |
| 4 | 12,3 | 0 | 9,0 |  |
| 5 | 12,4 | 0 | 0,0 |  |
| 6 | 12,5 | 0 | 1,9 | 12 |
| 7 | 12,6 | 0 | 3,3 | 12 |
| 8 | 12,7 | 0 | 3,6 |  |
| 9 | 12,9 | 0 | 1,9 | 12 |
| 10 | 13,9 | 0 |  |  |
| 11 | 13,9 | 0 | 1,2 | 8 |
| 12 | 13,9 | 0 | 1,2 | 8 |
| 13 | 14,3 | 0 | 2,1 |  |
| 14 | 14,4 | 0 | 0,0 |  |
| 15 | 14,4 | 0 | 2,4 | 12 |
| 16 | 14,7 | 0 | 0,0 | 12 |
| 17 | 14,7 | 0 | 2,0 | 12 |
| 18 | 14,8 | 0 | 0,0 | 12 |
| 19 | 14,9 | 0 | 1,1 |  |
| 20 | 15,4 | 0 | 0,0 | 12 |
| 21 | 15,8 | 0 | 1,9 | 12 |
| 22 | 15,9 | 0 | 3,0 | 12 |
| 23 | 16,0 | 0 | 4,0 | 12 |
| 24 | 17,2 | 0 | 1,9 | 12 |
| 25 | 17,4 | 0 | 2,1 | 12 |
| 26 | 17,5 | 0 | 0,3 |  |
| 27 | 17,6 | 0 | 0,3 | 12 |
| 28 | 17,6 | 0 | 2,2 | 12 |
| 29 | 19,8 | 0 | 10,0 | 8 |
| 30 | 19,9 | 0 |  |  |
| 31 | 25,5 | 0 |  |  |
| 32 | 0,1 | 1 | 8,6 | 4 |
| 33 | 0,6 | 1 | 12,2 | 4 |
| 34 | 1,0 | 1 |  | 8 |
| 35 | 1,1 | 1 | 15,2 | 6 |
| 36 | 1,3 | 1 | 0,0 | 8 |
| 37 | 1,4 | 1 | 2,9 | 8 |
| 38 | 1,5 | 1 | 2,5 | 12 |
| 39 | 1,5 | 1 | 15,4 | 9 |
| 40 | 1,6 | 1 | 3,0 | 9 |
| 41 | 1,7 | 1 | 30,6 | 4 |
| 42 | 1,8 | 1 | 2,7 | 12 |
| 43 | 2,0 | 1 | 2,9 | 8 |
| 44 | 2,0 | 1 | 18,8 | 0 |
| 45 | 2,0 | 1 | 26,7 | 6 |
| 46 | 2,4 | 1 | 1,7 | 8 |
| 47 | 2,5 | 1 | 1,1 | 8 |
| 48 | 2,6 | 1 | 2,6 | 8 |
| 49 | 2,8 | 1 |  | 12 |
| 50 | 2,8 | 1 | 3,6 | 4 |
| 51 | 3,0 | 1 | 2,2 | 8 |
| 52 | 3,0 | 1 | 7,7 | 6 |
| 53 | 3,0 | 1 | 10,8 | 12 |
| 54 | 3,1 | 1 | 6,5 | 6 |
| 55 | 3,2 | 1 | 3,6 | 8 |
| 56 | 3,4 | 1 | 6,5 | 8 |
| 57 | 3,4 | 1 | 6,8 |  |
| 58 | 3,6 | 1 | 3,0 | 8 |
| 59 | 3,7 | 1 | 4,2 | 8 |
| 60 | 3,9 | 1 | 3,3 | 8 |
| 61 | 3,9 | 1 | 3,7 | 4 |
| 62 | 4,2 | 1 | 4,2 | 12 |
| 63 | 4,3 | 1 | 10,0 | 4 |
| 64 | 4,4 | 1 | 3,8 | 8 |
| 65 | 4,4 | 1 | 4,1 | 12 |
| 66 | 4,4 | 1 | 4,9 | 8 |
| 67 | 4,4 | 1 | 9,5 | 8 |
| 68 | 4,5 | 1 | 2,5 | 8 |
| 69 | 4,5 | 1 | 3,6 | 12 |
| 70 | 4,7 | 1 | 11,4 | 4 |
| 71 | 4,9 | 1 | 0,0 | 8 |
| 72 | 4,9 | 1 | 1,8 | 8 |
| 73 | 5,0 | 1 | 2,0 | 8 |
| 74 | 5,1 | 1 | 2,7 | 8 |
| 75 | 5,2 | 1 |  |  |
| 76 | 5,2 | 1 | 2,7 | 12 |
| 77 | 5,3 | 1 | 1,4 | 8 |
| 78 | 5,4 | 1 | 2,5 | 12 |
| 79 | 5,8 | 1 | 1,6 | 12 |
| 80 | 6,1 | 1 | 1,9 | 12 |
| 81 | 6,1 | 1 | 11,2 | 8 |
| 82 | 7,3 | 1 | 1,7 |  |
| 83 | 7,5 | 1 | 2,7 | 8 |
| 84 | 7,7 | 1 | 6,7 | 8 |
| 85 | 7,9 | 1 | 2,7 | 12 |
| 86 | 8,2 | 1 | 18,0 | 12 |
| 87 | 8,3 | 1 | 3,4 | 8 |
| 88 | 8,5 | 1 | 3,2 | 8 |
| 89 | 9,0 | 1 | 1,3 | 12 |
| 90 | 9,2 | 1 | 4,6 | 12 |
| 91 | 9,4 | 1 | 1,0 | 8 |
| 92 | 9,4 | 1 | 6,3 | 8 |
| 93 | 9,5 | 1 |  | 8 |
| 94 | 9,7 | 1 | 7,5 | 8 |
| 95 | 10,0 | 1 | 2,8 | 12 |
| 96 | 10,4 | 1 | 2,0 |  |
| 97 | 10,4 | 1 | 15,5 | 8 |
| 98 | 10,9 | 1 | 10,3 | 12 |
| 99 | 11,4 | 1 | 1,8 |  |
| 100 | 11,9 | 1 | 1,4 | 8 |
| 101 | 11,9 | 1 | 3,2 | 12 |
| 102 | 12,1 | 1 | 1,6 | 8 |
| 103 | 12,7 | 1 | 4,9 |  |
| 104 | 17,8 | 1 |  | 12 |
| 105 | 18,1 | 1 | 3,4 | 12 |
| 106 | 18,2 | 1 | 0,0 | 12 |
| 107 | 0,0 | 2 | 0,7 | 8 |
| 108 | 0,0 | 2 | 1,8 | 8 |
| 109 | 0,1 | 2 | 2,6 | 12 |
| 110 | 0,2 | 2 | 0,0 | 12 |
| 111 | 0,2 | 2 | 1,1 | 8 |
| 112 | 0,2 | 2 | 14,3 | 4 |
| 113 | 0,3 | 2 | 6,1 | 6 |
| 114 | 0,4 | 2 | 0,5 | 12 |
| 115 | 0,4 | 2 | 1,1 |  |
| 116 | 0,4 | 2 | 28,6 | 2 |
| 117 | 0,5 | 2 | 2,2 | 12 |
| 118 | 0,5 | 2 | 34,9 | 6 |
| 119 | 0,6 | 2 | 2,4 | 12 |
| 120 | 0,6 | 2 | 3,3 | 12 |
| 121 | 0,9 | 2 | 1,3 | 12 |
| 122 | 1,0 | 2 | 1,5 | 8 |
| 123 | 1,0 | 2 | 4,0 | 8 |
| 124 | 1,0 | 2 | 4,5 | 12 |
| 125 | 1,1 | 2 | 0,0 | 12 |
| 126 | 1,1 | 2 | 3,3 | 12 |
| 127 | 1,2 | 2 | 3,8 |  |
| 128 | 1,2 | 2 | 11,1 |  |
| 129 | 1,4 | 2 | 0,0 | 8 |
| 130 | 1,4 | 2 | 0,9 | 12 |
| 131 | 1,4 | 2 | 1,5 | 8 |
| 132 | 1,4 | 2 | 1,9 | 12 |
| 133 | 1,4 | 2 | 3,6 |  |
| 134 | 1,5 | 2 | 1,1 | 12 |
| 135 | 1,6 | 2 | 0,0 | 9 |
| 136 | 1,6 | 2 | 0,0 | 8 |
| 137 | 1,6 | 2 | 4,6 | 12 |
| 138 | 1,7 | 2 |  | 12 |
| 139 | 1,7 | 2 | 1,9 | 12 |
| 140 | 1,7 | 2 | 3,5 | 12 |
| 141 | 1,8 | 2 | 0,0 | 12 |
| 142 | 1,9 | 2 | 0,0 |  |
| 143 | 1,9 | 2 | 0,9 | 8 |
| 144 | 1,9 | 2 | 3,2 |  |
| 145 | 1,9 | 2 | 5,7 |  |
| 146 | 2,1 | 2 | 0,0 | 12 |
| 147 | 2,2 | 2 | 0,4 | 12 |
| 148 | 2,2 | 2 | 3,1 | 8 |
| 149 | 2,3 | 2 | 0,8 | 8 |
| 150 | 2,3 | 2 | 3,4 | 12 |
| 151 | 2,3 | 2 | 8,1 | 7 |
| 152 | 2,4 | 2 | 0,0 | 12 |
| 153 | 2,5 | 2 | 4,7 | 6 |
| 154 | 2,6 | 2 | 0,0 | 12 |
| 155 | 2,6 | 2 | 0,0 | 12 |
| 156 | 2,7 | 2 | 0,0 | 12 |
| 157 | 2,7 | 2 | 1,9 | 12 |
| 158 | 2,9 | 2 | 0,0 |  |
| 159 | 2,9 | 2 | 3,0 | 8 |
| 160 | 3,1 | 2 | 1,4 | 12 |
| 161 | 3,1 | 2 | 5,2 | 4 |
| 162 | 3,1 | 2 | 5,7 | 6 |
| 163 | 3,2 | 2 | 0,6 |  |
| 164 | 3,2 | 2 | 0,7 | 12 |
| 165 | 3,2 | 2 | 1,7 |  |
| 166 | 3,2 | 2 | 2,5 | 8 |
| 167 | 3,2 | 2 | 3,8 | 12 |
| 168 | 3,3 | 2 | 0,7 | 12 |
| 169 | 3,4 | 2 | 0,0 |  |
| 170 | 3,4 | 2 | 6,7 | 12 |
| 171 | 3,6 | 2 | 0,4 | 12 |
| 172 | 3,6 | 2 | 2,1 | 12 |
| 173 | 3,6 | 2 | 2,6 | 12 |
| 174 | 3,8 | 2 | 0,0 | 8 |
| 175 | 3,8 | 2 | 0,2 | 12 |
| 176 | 3,8 | 2 | 1,3 | 12 |
| 177 | 3,8 | 2 | 1,9 | 9 |
| 178 | 3,9 | 2 | 0,0 | 12 |
| 179 | 3,9 | 2 | 0,0 |  |
| 180 | 3,9 | 2 | 0,0 |  |
| 181 | 3,9 | 2 | 0,9 | 12 |
| 182 | 4,0 | 2 | 1,5 | 8 |
| 183 | 4,0 | 2 | 20,5 | 2 |
| 184 | 4,1 | 2 | 0,0 | 12 |
| 185 | 4,2 | 2 | 1,9 | 12 |
| 186 | 4,2 | 2 | 5,1 | 9 |
| 187 | 4,3 | 2 |  |  |
| 188 | 4,4 | 2 | 1,1 | 12 |
| 189 | 4,5 | 2 |  | 12 |
| 190 | 4,5 | 2 | 0,0 |  |
| 191 | 4,5 | 2 | 2,6 | 12 |
| 192 | 4,5 | 2 | 4,6 | 8 |
| 193 | 4,6 | 2 |  | 8 |
| 194 | 4,6 | 2 | 0,6 | 8 |
| 195 | 4,7 | 2 | 5,4 | 12 |
| 196 | 4,8 | 2 | 0,5 | 12 |
| 197 | 4,8 | 2 | 3,7 | 8 |
| 198 | 4,9 | 2 | 2,4 | 8 |
| 199 | 4,9 | 2 | 4,6 | 12 |
| 200 | 5,0 | 2 |  |  |
| 201 | 5,1 | 2 | 0,0 | 12 |
| 202 | 5,1 | 2 | 0,6 | 12 |
| 203 | 5,1 | 2 | 0,8 |  |
| 204 | 5,1 | 2 | 1,2 | 12 |
| 205 | 5,1 | 2 | 13,6 | 12 |
| 206 | 5,2 | 2 | 0,6 | 12 |
| 207 | 5,4 | 2 | 1,8 |  |
| 208 | 5,6 | 2 | 2,1 | 12 |
| 209 | 5,6 | 2 | 2,4 | 8 |
| 210 | 5,7 | 2 | 3,6 | 8 |
| 211 | 5,9 | 2 | 1,1 | 12 |
| 212 | 5,9 | 2 | 2,1 |  |
| 213 | 6,0 | 2 | 3,6 | 12 |
| 214 | 6,1 | 2 | 0,9 | 12 |
| 215 | 6,1 | 2 | 5,1 |  |
| 216 | 6,2 | 2 | 0,3 | 4 |
| 217 | 6,2 | 2 | 3,7 | 12 |
| 218 | 6,2 | 2 | 8,4 | 12 |
| 219 | 6,4 | 2 |  | 12 |
| 220 | 6,4 | 2 | 3,1 | 8 |
| 221 | 6,5 | 2 | 7,9 |  |
| 222 | 6,6 | 2 | 0,0 | 12 |
| 223 | 6,8 | 2 | 3,1 | 12 |
| 224 | 7,0 | 2 |  |  |
| 225 | 7,0 | 2 | 7,5 | 12 |
| 226 | 7,2 | 2 | 2,1 | 12 |
| 227 | 7,2 | 2 | 2,7 | 12 |
| 228 | 7,3 | 2 | 0,0 |  |
| 229 | 7,3 | 2 | 0,7 | 8 |
| 230 | 7,4 | 2 | 1,0 | 12 |
| 231 | 7,6 | 2 | 0,0 | 12 |
| 232 | 7,6 | 2 | 13,2 |  |
| 233 | 7,8 | 2 | 0,0 | 12 |
| 234 | 7,9 | 2 | 9,6 | 12 |
| 235 | 8,0 | 2 | 1,0 | 12 |
| 236 | 8,0 | 2 | 1,2 | 12 |
| 237 | 8,0 | 2 | 3,2 | 12 |
| 238 | 8,3 | 2 | 1,1 | 8 |
| 239 | 8,4 | 2 | 5,9 | 12 |
| 240 | 8,5 | 2 | 0,0 | 12 |
| 241 | 8,6 | 2 | 0,2 | 8 |
| 242 | 8,6 | 2 | 1,3 |  |
| 243 | 8,6 | 2 | 4,1 | 8 |
| 244 | 8,7 | 2 | 3,0 |  |
| 245 | 8,9 | 2 | 0,9 |  |
| 246 | 8,9 | 2 | 1,7 | 8 |
| 247 | 9,0 | 2 | 8,8 | 12 |
| 248 | 9,1 | 2 | 0,0 | 12 |
| 249 | 9,4 | 2 | 4,2 | 4 |
| 250 | 9,5 | 2 | 0,4 | 12 |
| 251 | 9,5 | 2 | 1,8 | 8 |
| 252 | 9,5 | 2 | 2,3 | 8 |
| 253 | 9,6 | 2 | 1,0 | 12 |
| 254 | 9,6 | 2 | 1,5 | 12 |
| 255 | 9,7 | 2 |  |  |
| 256 | 9,8 | 2 | 1,3 | 12 |
| 257 | 9,8 | 2 | 2,5 | 12 |
| 258 | 9,9 | 2 | 1,7 | 12 |
| 259 | 10,0 | 2 | 0,0 | 12 |
| 260 | 10,0 | 2 | 0,3 | 12 |
| 261 | 10,0 | 2 | 0,7 | 12 |
| 262 | 10,2 | 2 | 0,0 | 8 |
| 263 | 10,2 | 2 | 1,1 | 12 |
| 264 | 10,3 | 2 | 1,7 | 12 |
| 265 | 10,6 | 2 | 0,0 | 12 |
| 266 | 10,8 | 2 | 4,2 | 8 |
| 267 | 10,9 | 2 | 0,5 | 12 |
| 268 | 10,9 | 2 | 0,5 | 12 |
| 269 | 11,3 | 2 | 0,3 | 12 |
| 270 | 11,6 | 2 | 2,1 |  |
| 271 | 11,7 | 2 |  |  |
| 272 | 11,9 | 2 | 0,0 |  |
| 273 | 11,9 | 2 | 2,5 | 8 |
| 274 | 12,0 | 2 | 1,0 |  |
| 275 | 12,0 | 2 | 1,4 | 8 |
| 276 | 12,0 | 2 | 1,8 | 12 |
| 277 | 12,1 | 2 |  | 12 |
| 278 | 12,1 | 2 | 2,9 |  |
| 279 | 12,1 | 2 | 2,9 |  |
| 280 | 12,4 | 2 | 0,6 | 12 |
| 281 | 12,4 | 2 | 1,0 | 8 |
| 282 | 12,8 | 2 | 0,0 | 12 |
| 283 | 12,9 | 2 |  |  |
| 284 | 13,4 | 2 | 3,3 | 8 |
| 285 | 13,5 | 2 | 7,5 | 8 |
| 286 | 13,9 | 2 |  | 12 |
| 287 | 13,9 | 2 | 0,0 |  |
| 288 | 13,9 | 2 | 1,0 | 12 |
| 289 | 13,9 | 2 | 1,0 |  |
| 290 | 14,0 | 2 | 0,0 |  |
| 291 | 14,1 | 2 | 0,0 | 12 |
| 292 | 14,3 | 2 | 2,8 | 8 |
| 293 | 14,5 | 2 | 2,2 | 12 |
| 294 | 15,0 | 2 |  |  |
| 295 | 15,6 | 2 | 5,5 |  |
| 296 | 15,7 | 2 | 3,2 | 12 |
| 297 | 16,1 | 2 | 3,9 |  |
| 298 | 16,2 | 2 | 0,0 | 12 |
| 299 | 16,2 | 2 | 18,5 | 12 |
| 300 | 16,4 | 2 | 0,6 |  |
| 301 | 17,5 | 2 | 2,5 | 12 |
| 302 | 17,9 | 2 | 4,6 | 12 |
| 303 | 18,1 | 2 | 2,7 | 8 |
| 304 | 21,1 | 2 | 1,8 |  |
| 305 | 2,0 | 3 | 0,0 | 8 |
| 306 | 5,8 | 3 | 3,5 | 6 |
| 307 | 5,8 | 3 | 4,8 | 8 |

IR= immunoreactivity score, for details pleases see material and methods.

Cancer specific survival: 0=alive, 1=death of prostate cancer,

2= death of other causes, 3= cause of death uncertain.
