# Supplementary material for: Immunoreactivity for prostate specific antigen and Ki67 differentiates subgroups of prostate cancer related to outcome
Source: Mod Pathol. 2019 Apr 12;32(9):1310–9. doi: 10.1038/s41379-019-0260-6 (PMC6760646; doi:10.1038/s41379-019-0260-6)
Supplement: Supplementary file 3 — table s2 [file 41379_2019_260_MOESM3_ESM.docx]

**Supplementary Table S2.** Number of patients and fraction of patients died from prostate cancer when differentiated based on the specified cut-off levels for Ki67 and PSA immunoreactivity (IR) as well as of the combinatory PSA/Ki67 IR scores derived thereof.

| **Cut-off IR scores** | **Events** | **Total** | **Fraction died** |
| --- | --- | --- | --- |
| **Ki67 med (> 2.7%)** |  |  |  |
| low | 22 | 169 | 0.13 |
| high | 48 | 117 | 0.41 |
| **Ki67 Q4 (> 5.4%)** |  |  |  |
| low | 48 | 241 | 0.20 |
| high | 22 | 45 | 0.49 |
| **PSA med (< 12)** |  |  |  |
| high | 21 | 139 | 0.15 |
| low | 48 | 108 | 0.44 |
|  |  |  |  |
| **Combinatory scores** |  |  |  |
| **PSA/Ki67 med** |  |  |  |
| high/low | 6 | 92 | 0.07 |
| high/high | 13 | 40 | 0.33 |
| low/low | 13 | 45 | 0.29 |
| low/high | 33 | 60 | 0.55 |
| **PSA/Ki67 Q4** |  |  |  |
| high/low | 16 | 121 | 0.13 |
| high/high | 3 | 11 | 0.27 |
| low/low | 28 | 78 | 0.36 |
| low/high | 18 | 27 | 0.67 |
