# Supplementary material for: Immunoreactivity for prostate specific antigen and Ki67 differentiates subgroups of prostate cancer related to outcome
Source: Mod Pathol. 2019 Apr 12;32(9):1310–9. doi: 10.1038/s41379-019-0260-6 (PMC6760646; doi:10.1038/s41379-019-0260-6)
Supplement: Supplementary file 4 — Suplemental figure legend [file 41379_2019_260_MOESM4_ESM.docx]

**Supplementary figure legend**

**Figure S1.** Sensitivity (black) and specificity (green) for Ki67 (a) and PSA (b) tumor immunoreactivity scores in identifying death from prostate cancer at different cut-off scores according to receiver operating characteristic curve analysis. Patients were diagnosed at transurethral resection of the prostate (1975-1991) and managed by watchful waiting. Median (PSA and Ki67) and quartile 4 (Ki67) levels for the cohort are indicated in red. The –log (*P*) values for Cox regression survival analysis using the indicated cut-off values are given in grey.
